# Did green debt instruments aid diversification during the COVID-19 pandemic?

**DOI:** 10.1186/s40854-021-00331-4

**Published:** 2022-03-03

**Authors:** Paresh Kumar Narayan, Syed Aun R. Rizvi, Ali Sakti

**Affiliations:** 1 Monash University, Melbourne, Australia; 2 Lahore University of Management Sciences, Lahore, Pakistan; 3 Bank Indonesia, Jakarta, Indonesia

**Keywords:** COVID-19, Green Sukuk, Indonesia Capital market, G11, G15, G19

## Abstract

Faced with a persistent pandemic, investors are concerned about portfolio diversification. While the literature on COVID-19 has evolved impressively, limited work remains on diversification opportunities. We contribute to the literature by exploring the volatility and co-movement of different sovereign debt instruments, including green sukuk, sukuk, bond and Islamic and conventional equity indices for Indonesia. Our results consistently point towards increased asset co-movement and weak profitability during the pandemic. Interestingly, sukuk and green sukuk have a 14% correlation with stocks, suggesting potential diversification prospects in times of extreme shocks.

## Introduction

Global crises, whether financial or economic, bring to attention the role of portfolio diversification. The literature on portfolio diversification in times of crises is rich and perceives debt instruments as attractive tools (see, *inter alia*, Boucher and Tokpavi 2019; Selmi et al. 2019; Skintzi 2019). The current COVID-19 pandemic, because it is more persistent than any previous crisis, has had a dynamic effect on the financial system. In other words, the effects have been experienced in stages. The initial stage, for instance, was one when the pandemic started and markets overreacted; then, with time, as more was understood about the pandemic, markets corrected their overreaction (Harjoto et al. 2021). In this regard, many studies have demonstrated how equity markets (see, *inter alia*, Haroon and Rizvi 2020a,b; Narayan 2020a; Sharma 2020) and energy markets (see, for instance, Iyke 2020; Polemis and Soursou 2020; Gil-Alana and Monge 2020) globally have reacted to the pandemic over time. Some studies have also explored the interaction between different asset classes: exchange rates (Narayan 2020b), oil and exchange rates (Devpura 2020), stocks and exchange rates (Prabheesh and Kumar 2021), stocks and bonds (Papadamou et al. 2020) and cryptocurrencies (Yousaf and Ali 2020; Shahzad et al. 2021).^1^

The literature alluded to above contains an important gap: limited studies have considered diversification prospects in light of the pandemic from a green investment^2^ point of view. In this study, we explore the potential diversification prospects. We argue that faced with the pandemic, investors are likely to behave consistent with the flight-to-quality phenomena. In this pandemic situation, we perceive investors as treating market volatility and connectedness between sovereign debt and equity markets differently (that is, by considering the dynamic and persistent nature of the pandemic). When diversification prospects are evaluated, a multi-asset class model is suitable. We, therefore, consider an asset class that includes Islamic debt and equity markets. Over the years, Islamic finance has developed into a unique asset class; see Narayan and Phan (2019) for a survey.^3^

A relatively recent innovation in the debt market is the green debt instruments. An attractive feature of this instrument has been its use in financing renewable energy projects globally (see, *inter alia*, Tang and Zhang 2020; Banga 2019; Hachenberg and Schiereck 2018). However, the role of the debt market in an asset diversification portfolio needs further inquiry given the ramifications of the COVID-19 pandemic.

To explore the volatility and inter-connectedness of multiple asset classes within stocks and debt during the COVID-19 pandemic, we focus on the conventional stock market (IDX Composite/Indeks Harga Saham Gabungan), its Islamic counterpart (Jakarta Islamic Index), the conventional debt market (sovereign bond), its Islamic counterpart (sovereign sukuk), and the green sukuk from Indonesia. Our hypothesis is that the interplay between these asset classes has evolved and changed owing to the pandemic. Our hypothesis is motivated by earlier work on previous pandemics by Bhuyan et al. (2010), who highlight that stock market returns of the infected countries exhibit a significant increase in co-movements. The evolving recent literature shows that pandemic has impacted asset prices substantially differently during the COVID-19 period. Amongst these studies, Iyke (2020) shows not only that the pandemic predicts asset prices but how its influence changed during the pandemic.^4^ Focusing on the exchange rate market, Narayan (2020a) shows how bubble activity intensified, and how the exchange rate became more resilient to shocks during the pandemic. Moreover, Narayan (2020b) shows how the oil market return/volatility changed due to the pandemic. In addition, Prabheesh and Kumar (2021) show that exchange rate remains neutral while energy and financial markets were affected by COVID uncertainty.^5^ Other studies offer equally important insights: Appiah-Otoo (2020), for instance, reveal using Chinese data that exchange rate significantly reduces domestic credit during the pandemic; Salisu and Sikiru (2020) find that during the COVID-19 period uncertainty of the pandemic is a factor for Asia–Pacific Islamic stock returns; and Qin et al. (2020) show that the pandemic has a negative effect on the oil price. Overall, we conclude from the literature that the impact of the recent pandemic on financial markets has exacerbated uncertainty—a point demonstrated by Sharma (2020). This literature, therefore, inspires the following questions. What has happened to asset price correlations over time including over the pandemic period? Has this portfolio diversification or otherwise influenced profits from those asset classes we consider? Has there been a flight to quality because of the uncertainty created by the pandemic? Our hypothesis allows us to address these questions and contribute to our understanding of asset pricing behavior in the COVID-19 pandemic period.

Our focus on Indonesia has roots in its unique structure and diverse set of financial asset class offerings. As highlighted by Sharma et al. (2019), Indonesia is uniquely poised as its equity market is large yet underdeveloped, offering opportunities for growth. As the fourth largest global population, the Indonesian market boosts of a range of diverse products across both conventional and Islamic asset classes. Indonesia also boosts the largest Muslim population in the world offering a range of Islamic products. Indonesia is also a leader in sovereign issuance of green sukuk.^6^ These features make a study on Indonesia’s asset co-movement from the point of view of diversification benefits ideal.

We test our hypothesis on the evolution of risks and returns from multiple two-asset portfolios by using daily data (March 4, 2019 to December 4, 2020) fitted to a multivariate GARCH model. To evaluate the impact of the COVID-19 pandemic, the sample is divided into a pre-COVID-19 and a COVID-19 sub-sample periods. Our findings and contributions are as follows. We show that the volatility of all assets increased during the COVID-19 pandemic. While this finding adds to the evolving literature on financial market volatility during COVID-19 (see Haroon and Rizvi 2020a, b; Ali et al. 2020; Salisu and Adediran 2020), the insights we provide come from a unique set of assets that include less riskier assets, such as sukuk and green sukuk, which have not been subjected to this type of empirical investigation before.

We, therefore, explore the correlations between asset pairs consisting of conventional and Islamic stocks, bonds, and sukuk and green sukuk. By considering a mixture of risky and less risky assets, we offer new insights on the dynamic relationship among assets that have implications for portfolio formation and risk diversification. We find high correlations: averaging over 80% between stocks, and these correlations increased during the pandemic. Both sukuk and green sukuk have low correlations with stocks and although these correlations increase in the pandemic period, they remain low, at less than 14%. The implication is that both sukuk and green sukuk offer diversification benefits during the pandemic. When we utilize a two-asset portfolio consisting of the same pairs of assets used to obtain correlations, we find, in general, that diversification offers greater profits from a two-asset portfolio weight optimization model. Our findings support the mixed evidence in the literature on green debt instruments. This literature (see, for instance, Nguyen et al. 2021; Reboredo et al. 2020) finds evidence of: (a) low correlations between green bonds and commodities; and (b) price spillover from fixed income to green bonds. We add to this by showing potential diversification benefits when it comes to sukuk and green sukuk.

Finally, because in our exercise, we have both risky assets (such as the conventional and Islamic stocks) and less risky assets (such as bonds, sukuk and green sukuk), this allows us to test the flight to quality hypothesis—the idea that during crises investors prefer holding less risky assets. If this is true, then we should expect to see a switch from risky assets to less risky assets. Using a Granger causality test, we find strong evidence supporting Granger causality from Islamic and conventional stocks to sukuk and green sukuk. The evidence is, as expected, much stronger during the COVID-19 pandemic period.

Lastly, the COVID-19 pandemic has instigated a rich literature on the effects of the pandemic on the financial system and asset prices (see, *inter alia*, Yan and Qian 2020; Sharma 2020; Iyke 2020; Sha and Sharma 2020; Sharma and Sha 2020). In this literature, none of these studies has yet analyzed how sukuk and green sukuk are connected to stocks and bonds from a portfolio diversification perspective. By doing so, we provide additional insights on how asset connectedness has evolved in light of the pandemic. In addition, our study contributes to the evolving literature on sovereign debt classes and equity nexus which concludes with mixed results (see Samour et al. 2020; Golab et al. 2018; Allegret et al. 2017).

We engage in robustness tests to confirm our key findings. Our attempt starts with sample splitting. We decompose the full sample into a pre-COVID pandemic sub-sample, an epidemic sub-sample (before the COVID-19 was declared a pandemic), and a pandemic sub-sample owing to the date on which the World Health Organization (WHO) declared COVID-19 a pandemic. We consistently find evidence of low correlations between sukuk/green sukuk vis-à-vis other assets. Secondly, when we localize the date of the start of COVID-19 to Indonesia and create sub-samples, which are Indonesia-specific, our main results remain insensitive. Thirdly as an alternative econometric model, we employ the exponential GARCH model to estimate volatility for our data sample. Results from this exercise do not change our story.

## Data and methodology

Given that the COVID-19 pandemic is a recent crisis, the data are limited. For instance, the first confirmed case was reported on December 31, 2019. In this study, we use Indonesian data: namely, Indonesia’s sovereign 5-year debt instruments (bond, sukuk and green sukuk). For equities, we use the benchmark conventional stock market index (IDX Composite/ Indeks Harga Saham Gabungan) and the Islamic index from the Jakarta stock exchange (Jakarta Islamic Index).

The data spans March 4, 2019 to December 4, 2020, which gives us 225 and 240 observation (in terms of days) for pre and post the first COVID-19 reported case on December 31, 2020.^7^ We also segregate the COVID-19 period into a pre-COVID-19 (March 4, 2019 to December 30, 2019) sample. We create a sub-sample marked by the period when the WHO had not declared COVID-19 a pandemic; we refer to this as an epidemic phase (December 31, 2019 to March 10, 2020). Finally, we have a pandemic sub-sample covering the time from when the WHO declared COVID-19 as a pandemic (March 11, 2020 to December 4, 2020). In additional analysis, we categorize the pre-COVID-19 and post-COVID-19 timelines localized specifically to the Indonesian context given that the first reported case in Indonesia was on March 2, 2020.^8^ With this date, for Indonesia, we have a pre-COVID-19 period from March 4, 2019 to March 1, 2020 and a COVID-19 period from March 2, 2020 to December 4, 2020.

Daily returns for all the sample are calculated using the equation, ${r}_{t}={ln(P}_{t})-{ln(P}_{t-1})$*.* Here, ${r}_{t}$ and ${P}_{t}$ denote daily returns and price at the business day *t,* respectively, and *ln* represents the natural log.

To study the volatility and correlations of the sovereign bond, sukuk, green sukuk, Islamic stocks, and conventional stocks, we employ a Multivariate Generalized Autoregressive Conditional Heteroscedastic-Dynamic Conditional Correlation (MGARCH-DCC) model proposed by Engle (2002) and Pesaran and Pesaran (2009). The MGARCH-DCC is suitable to obtain the variance and correlations between assets over time. This method is popular and widely used in applications, and can be stated as:

1 $${r}_{t}= {\mu }_{t}+{\varepsilon }_{t}$$

where ${\mu }_{t}=\mathrm{\rm E}\left[{r}_{t\left|{\Omega }_{t-1}\right|}\right]$, ${\mu }_{t}|{\Omega }_{t-1}\sim N(0, {H}_{t})$, ${H}_{t}={D}_{t}{R}_{t}{D}_{t}$, ${D}_{t}=diag\{\sqrt{{h}_{ii.t}}\}$, and ${z}_{t}={D}_{t}^{-1}{\varepsilon }_{t}$. In this, ${h}_{ii.t}$ is the estimated conditional variance from the individual univariate GARCH model; ${D}_{t}$ is the diagonal matrix of conditional standard deviations; ${R}_{t}$ is the time-varying conditional correlation coefficient matrix of returns; and ${z}_{t}$ is the standardized residuals vector with mean zero and variance one. The dynamic correlation coefficient matrix of the DCC model can be specified further as per Hsu et al. (2008):

2 $${R}_{t}=\left({diag\left({Q}_{t}\right)}^{-1/2}{Q}_{t}({diag\left({Q}_{t}\right))}^{-1/2}\right)$$

where ${Q}_{t}=({q}_{ij,t})$ and $({diag\left({Q}_{t}\right))}^{-1/2}= \left(diag\frac{1}{\sqrt{{q}_{11,t}}},\dots ,\frac{1}{\sqrt{{q}_{nn,t}}}\right)$ with ${q}_{ij,t}={\overline{\rho }}_{ij}+a\left({z}_{i, t-1}{z}_{j,t-1}-{\overline{\rho }}_{ij}\right)+\beta ({q}_{ij,t-1}-{\overline{\rho }}_{ij})$ in which ${\overline{\rho }}_{ij}$ is the unconditional correlations and the new time-varying conditional correlation coefficient is ${\rho }_{ij,t}={q}_{ij,t}/\sqrt{{q}_{ii,t}{q}_{jj,t}} .$

## Analysis

### Descriptive statistics

Table 1 provides descriptive statistics of the five assets. We report average returns and standard deviation of the data across various sub-samples. Panel A provides the descriptive statistics for the pre-COVID-19 period (March 4, 2019 to December 30, 2019) and the COVID-19 phase (December 31, 2019 to December 4, 2020). Panel B presents corresponding descriptive statistics for the pre-COVID period (March 4, 2019 to December 30, 2019), the epidemic phase (December 31, 2019 to March 10, 2020), and the pandemic phase (March 11, 2020 to December 4, 2020). Panel C defines pre-COVID-19 and COVID-19 periods specifically for Indonesia based on the first reported COVID-19 case in Indonesia as discussed earlier.^9^ Consistent with recent studies (see those in Sha and Sharma (2020)(2020 and Narayan et al. (2020), for instance), returns in the pandemic period have been relatively low with higher variance.

**Table 1:** Descriptive statistics

			Panel A		Panel B			X`	
		Full Sample	Pre-COVID	COVID-Time	Pre-COVID	Epidemic	Pandemic	Pre-COVID (Indonesia)	COVID-Time (Indonesia)
Bonds	Mean	0.0038%	0.0065%	0.0016%	0.0065%	0.0199%	− 0.0033%	0.0066%	0.0010%
	St. Dev	0.0011	0.0011	0.0010	0.0011	0.0010	0.0010	0.0011	0.0000
Sukuk	Mean	0.0197%	0.0197%	0.0197%	0.0197%	0.0312%	0.0165%	0.0227%	0.0165%
	St. Dev	0.0028	0.0016	0.0035	0.0016	0.0021	0.0038	0.0016	0.0002
Green sukuk	Mean	0.0208%	0.0271%	0.0155%	0.0271%	− 0.0097%	0.0223%	0.0260%	0.0154%
	St. Dev	0.0019	0.0009	0.0025	0.0009	0.0016	0.0027	0.0008	0.0002
Conventional stock	Mean	− 0.0236%	− 0.0123%	− 0.0331%	− 0.0123%	− 0.3859%	0.0624%	− 0.0366%	− 0.0100%
	St. Dev	0.0130	0.0072	0.0164	0.0072	0.0139	0.0169	0.0072	− 0.0001
Islamic stocks	Mean	− 0.0299%	− 0.0044%	− 0.0514%	− 0.0044%	− 0.4856%	0.0662%	− 0.0398%	− 0.0195%
	St. Dev	0.0162	0.0100	0.0200	0.0100	0.0171	0.0206	0.0099	− 0.0002

This table presents the mean and standard deviations of the daily bond, sukuk, green sukuk, conventional stock index and Islamic stock index returns from Indonesia. First column provides the average values for the full sample of data, from March 4, 2019 to December 4, 2020. Panel A provides descriptive statistics for the pre-COVID-19 period (March 4, 2019 to December 30, 2019) and the COVID-19 period (December 31, 2019 to December 4, 2020). Panel B presents descriptive statistics for the pre-COVID-19 period (March 4, 2019 to December 30, 2019), the epidemic phase of COVID-19 (December 31, 2019 to March 10, 2020) and the pandemic phase (March 11, 2020 to December 4, 2020). Panel C defines the pre-COVID-19 and the COVID-19 periods specific to Indonesia, where the pre-COVID-19 phase is from March 4, 2019 to March 1, 2020 and the COVID-19 phase is from March 2, 2020 to December 4, 2020

The data suggest that the sovereign sukuk issued by the Government of Indonesia is least affected in terms of average returns during the pandemic. We also observe that in the case of green sukuk, there was a sharp decline in returns during the epidemic phase but returns recovered during the pandemic phase. The implication is that because asset price reaction to the different phases of COVID-19 has been heterogeneous (see Sharma and Sha 2020), the conditional correlations and volatilities are likely to be heterogeneous.

### Correlations

To formally test this, we turn to Tables 2 and 3 where average conditional volatility and conditional correlations for different asset combinations and sub-samples are presented. From results described in Table 2, we observe that over the full sample of data across the five assets, Islamic stocks were most volatile followed by conventional stocks. Bonds were least volatile together with green sukuk. Panel A tells that except for bonds, which became least volatile in the COVID-19 period, all other assets were more volatile in the COVID-19 period compared to the pre-COVID-19 period. This is true regardless of how we define COVID-19 dates (see Panel C). Panel B suggests that even when COVID-19 was not declared a pandemic—that is, at the epidemic stage, three of the five assets had higher volatility compared to the pre-COVID-19 period. In this regard, we see that bonds and green sukuk volatility was less sensitive to the epidemic. Overall, the largest increase in volatility is noted for stocks and least for sukuk-based assets.

**Table 2:** Average sample volatility of assets

	Full Sample	Panel A		Panel B			Panel C	
		Pre-COVID	COVID-Time	Pre-COVID	Epidemic	Pandemic	Pre-COVID (Indonesia)	COVID-Time (Indonesia)
Bond	0.0010	0.0011	0.0009	0.0011	0.0009	0.0009	0.0011	0.0009
Sukuk	0.0021	0.0015	0.0026	0.0015	0.0021	0.0028	0.0016	0.0027
Green sukuk	0.0013	0.0009	0.0016	0.0009	0.0005	0.0019	0.0008	0.0018
Conventional stock	0.0108	0.0070	0.0139	0.0070	0.0086	0.0154	0.0069	0.0149
Islamic stocks	0.0136	0.0098	0.0168	0.0098	0.0114	0.0183	0.0096	0.0178

This Table provides the average of conditional volatilities of the five asset returns; namely bonds, sukuk, green sukuk, conventional stocks, and Islamic stocks. First column provides the average values for the full sample of data, from March 4, 2019 to December 4, 2020. Panel A provides descriptive statistics for the pre-COVID-19 period (March 4, 2019 to December 30, 2019) and the COVID-19 period (December 31, 2019 to December 4, 2020). Panel B presents descriptive statistics for the pre-COVID-19 period (March 4, 2019 to December 30, 2019), the epidemic phase of COVID-19 (December 31, 2019 to March 10, 2020) and the pandemic phase (March 11, 2020 to December 4, 2020). Panel C defines the pre-COVID-19 and the COVID-19 periods specific to Indonesia, where the pre-COVID-19 phase is from March 4, 2019 to March 1, 2020 and the COVID-19 phase is from March 2, 2020 to December 4, 2020

**Table 3:** Average conditional correlations amongst asset pairs

	Full Sample (%)	Panel A				Panel B			Panel C		
		Pre-COVID-19 (%)	*t*-test between Full Sample & Pre-COVID-19	COVID-19 (%)	*t*-test between Pre-COVID & COVID-19	Epidemic (%)	Pandemic (%)	*t*-test between Epidemic & Pandemic	Pre-COVID-19 (Indonesia) (%)	COVID-19 (Indonesia) (%)	*t*-test between Pre-COVID & COVID-19
Sukuk & Bond	13.99	11.64	− 0.55	15.97	− 7.89	6.23	18.60	− 30.09	10.91	17.20	− 6.31
Green Sukuk & Bond	15.50	16.12	− 7.78	14.97	1.00	3.54	18.07	− 6.89	14.29	16.76	− 1.52
Green Sukuk & Sukuk	39.00	51.21	− 3.05	28.69	21.67	16.28	32.06	− 13.13	47.33	30.28	13.40
Conventional Stock & Bond	19.47	16.27	− 0.83	22.18	− 11.38	10.97	25.22	− 33.69	15.00	24.16	− 13.07
Conventional Stock & Sukuk	2.19	− 5.31	0.33	8.52	− 12.92	− 4.52	12.06	− 22.12	− 5.09	9.80	− 14.00
Conventional Stock & Green Sukuk	11.50	11.39	− 4.75	11.60	− 0.55	− 8.62	17.08	− 18.82	9.33	13.78	− 2.73
Islamic Stock & Conventional Stock	85.69	76.39	8.02	93.54	− 15.11	92.13	93.92	− 14.91	77.83	93.90	− 13.90
Islamic Stock & Bond	17.76	14.99	− 1.17	20.10	− 8.54	6.06	23.90	− 34.48	13.51	22.20	− 9.14
Islamic Stock & Sukuk	− 0.10	− 9.07	0.69	7.47	− 14.06	− 6.28	11.20	− 27.18	− 8.79	8.98	− 15.12
Islamic Stock & Green Sukuk	8.30	8.31	− 6.05	8.28	− 0.37	− 14.00	14.32	− 22.13	5.92	10.78	− 2.85

This table provides the average of conditional correlations amongst asset return pairs. The null hypothesis that the correlations are zero is also tested and the resulting t-tests are reported. The first column provides the average correlations over the full sample (March 4, 2019 to December 4, 2020). Panel A provides the sample average conditional correlations for the pre-COVID-19 period (March 4, 2019 to December 30, 2019) and the COVID-19 period (December 31, 2019 to December 4, 2020). Panel B presents the sample average conditional correlations for the epidemic phase of COVID-19 (December 31, 2019 to March 10, 2020) and the pandemic phase (March 11, 2020 to December 4, 2020). Panel C defines pre-COVID-19 and COVID-19 period specific to the Indonesian case, where the pre-COVID phase is from March 4, 2019 to March 1, 2020 and the COVID-19 phase is from March 2, 2020 to December 4, 2020. The first three rows of the table correlations are conditional correlations for pairs of debt securities. The next three rows contain conditional correlations for conventional stocks with bonds, sukuk, and green sukuk. The last four rows have conditional correlations of Islamic stocks with conventional stocks, bonds, sukuk and green sukuk

Table 3 presents average conditional correlations. The correlations are divided into three parts. The first part of table provides conditional correlations for pairs of debt securities. The second part presents the conditional correlations of conventional stocks with bonds, sukuk, and green sukuk. The third part presents the conditional correlations of Islamic stocks with conventional stocks, bonds, sukuk and green sukuk. Starting with sukuk, green sukuk and bond correlations, we see that over the full sample, sukuk-green sukuk had the highest correlation at 39%. In the pre-COVID-19 period, it had the highest correlation at 51%. In the COVID-19 phase, this correlation declined to around 30% (Panel C). The sukuk-bond correlations were lowest, from around 14% in the full-sample to around 11% in the pre-COVID-19 sample (Panel C). It was the least at 3.54% during the epidemic stage (Panel B). Overall, in the COVID-19 phase sukuk-bond correlations have increased compared to the pre-COVID-19 phase. The exception is the correlation between green sukuk and sukuk, which declined in the COVID-19 phase.

The second part of the correlations between conventional stocks and bond/sukuk suggests that correlations have grown in the COVID-19 phase compared to the pre-COVID-19 times. The weakest correlations though are for conventional stocks and sukuk followed by conventional stocks and green sukuk.

To the last set of results on Islamic stocks, conventional stocks, bonds, sukuk, and green sukuk, we see that stock level correlations are highest amongst all pairs and has increased substantially over the COVID-19 period (average of over 93%) compared to around 80% average correlation in the pre-COVID-19 period. Islamic stock–bond correlation has also increased in the COVID-19 period, but it is around 20%. Islamic stocks and sukuk are weakly correlated both before and during the pandemic; however, in the COVID-19 phase, average correlation has risen to as much as 11%. A similar pattern is observed for the correlation between Islamic stocks and green sukuk with correlations maximizing at 14% during the pandemic. This suggests flight-to quality phenomena, as discussed by Papadamou et al. (2020), regarding COVID-19’s impact on financial asset classes.

### A test of the flight to quality hypothesis

As we discussed in the introduction, our test of pair-wise correlations between asset classes was motivated by the flight to quality hypothesis: that investors prefer less risky assets during crises compared to riskier assets. One way of testing this would be examine if trading volume in risky assets, such as equity, increased during the COVID-19 pandemic. We are unable to test this given that trading volume data for those asset classes in our sample are unavailable. As an alternative, we employ a Granger causality test^10^ based on a VAR model with four lags. Our motivation for using a VAR model is as follows. If the riskier assets in our portfolio—namely, conventional and Islamic stocks—Granger cause less riskier assets—namely, bond, sukuk and/or green sukuk—this is evidence of flight to quality phenomena at work. To set the scene of how the assets have Granger caused each other, we start with results in Panel A of Table 4, based on the full sample of data. We see evidence of Granger causality running from conventional and Islamic stocks to green sukuk. When we explore the pre-COVID-19 flight to quality hypothesis with the COVID-19 phase, we find stronger evidence of flight to quality. That is, during the COVID-19 period, conventional and Islamic stocks Granger cause both sukuk and green sukuk. By comparison, in the pre-COVID-19 period, conventional and Islamic stocks only Granger caused green sukuk. Our work in this regard is preliminary and should be open to additional assessment of capital flight.

**Table 4:** Granger Causality

*Panel A – Pre-COVID*						*Panel B – COVID-19*				
Dependent Variable	*Bonds*	*Sukuk*	*Green Sukuk*	*Conventional Stock*	*Islamic Stocks*	*Bonds*	*Sukuk*	*Green Sukuk*	*Conventional Stock*	*Islamic Stocks*
*Bonds*		4.1662	7.7015*	2.1116	0.51263		11.268**	37.038***	3.1416	5.2473
		(0.244)	(0.053)	(0.550)	(0.916)		(0.010)	(0.000)	(0.370)	(0.155)
*Sukuk*	4.9806		20.639***	4.6605	3.8434	6.1361		280.31***	4.5941	5.8249
	(0.173)		(0.000)	(0.198)	(0.279)	(0.105)		(0.000)	(0.204)	(0.120)
*Green Sukuk*	0.93114	3.2549		6.1314	4.8424	9.6376*	38.768***		4.7567	6.1779
	(0.818)	(0.354)		(0.105)	(0.184)	(0.022)	(0.000)		(0.191)	(0.103)
*Conventional Stock*	3.172	5.551	10.105**		4.1254	0.83493	6.2745*	37.63***		6.0587
	(0.366)	(0.136)	(0.018)		(0.866)	(0.841)	(0.099)	(0.000)		(0.170)
*Islamic Stocks*	5.2739	5.7914	7.408*	3.311		2.3226	7.9474**	35.145***	4.9063	
	(0.153)	(0.122)	(0.060)	(0.346)		(0.508)	(0.047)	(0.000)	(0.179)	

This table provides the Granger causality WALD test results for the five financial assets under discussion. The table presents the chi2 value and and p value in parenthesis for each granger causal relation. Panel A provides the VAR Granger causality WALD test for the pre-COVID-19 period (March 4, 2019 to March 1, 2020) and Panel B provides VAR Granger causality WALD test for the COVID-19 period (March 2, 2020 to December 4, 2020). First column presenting the dependent variable. *, **, *** denote significance at 10%, 5%, 1% respectively

### Economic significance of correlations

The objective of this sub-section is to explore, for each pair of our asset portfolio, profits based on obtaining a portfolio weight that is dynamic by construction. The idea is to see whether high asset correlations do indeed deliver less profits. In other words, the more diversified the portfolio (that is, the lower the correlations), the higher the profit. To achieve this goal, we draw on the two-asset portfolio weight optimization of Kroner and Ng (1998). To see how this weight is obtained, consider one of our pair of assets, namely Sukuk and bond. In this case, the weight $\left({w}_{t}\right)$ of the Sukuk market in a one-dollar portfolio of Sukuk and bond (US) at time *t* is given by:

3 $${w}_{t}=\frac{{h}_{t}^{Bond}-{h}_{t}^{Bond,Sukuk}}{{h}_{t}^{Sukuk}-2{h}_{t}^{Sukuk, Bond}+{h}_{t}^{Bond}}$$

The time-varying conditional variance of the bond market $\left({h}_{t}^{Sukuk}\right)$ and the bond market $\left({h}_{t}^{Bond}\right)$, and the conditional covariance $\left({h}_{t}^{Sukuk,Bond}\right)$ are extracted from estimating a bivariate GARCH model.

Two trends are worth a discussion: (1) we see that low-risk asset portfolios, in general, have lower profits (between 2 to 7% in the COVID-19 pandemic period) compared to when one of the two assets in the portfolio is a riskier asset, such as the Islamic stock–bond portfolio (8.24%) and Islamic stock-sukuk portfolio (11.27%); and (2) in general, profits are higher for portfolios of lower correlations (see Table 5).

**Table 5:** Economic significance results

	Pre-COVID-19	COVID-19
Sukuk & Bond	5.95%	2.59%
Green Sukuk & Bond	6.36%	2.58%
Green Sukuk & Sukuk	8.88%	6.16%
Conventional Stock & Bond	− 9.61%	6.26%
Conventional Stock & Sukuk	− 5.67%	9.80%
Conventional Stock & Green Sukuk	− 6.38%	9.37%
Islamic Stock & Conventional Stock	− 24.75%	14.66%
Islamic Stock & Bond	− 12.39%	8.24%
Islamic Stock & Sukuk	− 12.27%	11.76%
Islamic Stock & Green Sukuk	− 9.08%	12.37%

This table provides the optimized return which minimizes risk without reducing expected return as defined by Kroner and Ng (1998). The pre-COVID phase is from March 4, 2019 to March 1, 2020 and the COVID-19 phase is from March 2, 2020 to December 4, 2020

A final observation is that the 10-asset portfolios we consider are all profitable in the COVID-19 period, with annualized profits in the 2.59% (sukuk-bond) to 14.66% (conventional stock-Islamic stock) range. This is consistent with the stronger performance of the financial market in the aftermath of the early market panic due to the pandemic (see Sha and Sharma 2020 and Sharma and Sha 2020 for a summary of the literature).

### Robustness test

One concern with our correlation and flight to quality hypothesis is that in taking returns we have not adjusted for other potential risk factors. We do this now. We proceed in two steps. In the first step we regress excess returns (on each asset) on excess market returns, stock price volatility, exchange rate, and day-of-the-week dummies variables. For further details, see notes to Table 6 where the results for the flight to capital hypothesis is reported. We see that our main finding is insensitive to the adjustment of potential market and macro risk factors. The flight to capital holds strongly during the COVID-19 phase. Not only Islamic (conventional) stocks Granger cause bonds and sukuk (bonds and green sukuk) but bonds and sukuk Granger cause green sukuk.

**Table 6:** Robustness Test

*Panel A – Pre-COVID*						*Panel B – COVID-19*				
Dependent Variable	*Bonds*	*Sukuk*	*Green Sukuk*	*Conventional Stock*	*Islamic Stocks*	*Bonds*	*Sukuk*	*Green Sukuk*	*Conventional Stock*	*Islamic Stocks*
*Bonds*		1.1499	2.3452	67.904***	0.4654		1.2852	6.794**	46.679***	0.66955
		(0.563)	(0.310)	(0.000)	(0.792)		(0.526)	(0.033)	(0.000)	(0.715)
*Sukuk*	0.3423		3.1707	18.759***	1.938	0.0422		80.411***	1.0233	8.0294**
	(0.843)		(0.205)	(0.000)	(0.379)	(0.979)		(0.000)	(0.599)	(0.018)
*Green Sukuk*	0.2237	1.6002		32.047***	5.0952*	1.1735	0.129		13.852***	4.7568*
	(0.894)	(0.449)		(0.000)	(0.078)	(0.556)	(0.938)		(0.001)	(0.093)
*Conventional Stock*	0.1483	3.9826	0.26059		4.5787	5.3209*	13.796***	6.7266**		3.2755
	(0.929)	(0.137)	(0.878)		(0.101)	(0.070)	(0.001)	(0.035)		(0.194)
*Islamic Stocks*	2.4464	0.35056	3.802	10.545***		5.728*	5.7216*	0.6077	8.5643**	
	(0.294)	(0.839)	(0.149)	(0.005)		(0.057)	(0.057)	(0.738)	(0.014)	

This table provides the results for robustness test using time series adjusted returns. Panel A provides the VAR Granger causality WALD test for the pre-COVID-19 period (March 4, 2019 to March 1, 2020) and Panel B provides VAR Granger causality WALD test for the COVID-19 period (March 2, 2020 to December 4, 2020). First column presenting the dependent variable. *, **, *** denote significance at 10%, 5%, 1% respectively. Two steps are performed. In the first step, time series adjusted returns have been calculated based on the regression equation: ${ER}_{t}=\alpha +{\beta }_{1}{Mkt}_{t}+{\beta }_{2}{Ex}_{t-1}+{\beta }_{3}{Vol}_{t}+{\beta }_{4}{Day}_{t}+{\varepsilon }_{t}$ where ${ER}_{t}$ is excess returns (raw returns in excess of the short rate), ${Mkt}_{t}$ is the excess market return (which we proxy using the Jakarta stock exchange price), ${Ex}_{t-1}$ is the bilateral exchange rate (Rupiah vis-à-vis the US dollar), $Vol$ is the stock market return volatility and $Day$ is the day-of-the-week effect, where Monday, Tuesday, Thursday and Friday dummies are used

## Concluding remarks

The COVID-19 pandemic has brought to attention the issue of portfolio diversification. This is also an issue for emerging markets such as Indonesia. In this paper, we focus on Indonesia given its progress on sukuk and green sukuk as financial instruments. We explore the volatility and correlation patterns in Indonesia’s sovereign bond, its stock market (both conventional and Islamic markets), sukuk and green sukuk. In evaluating these five asset classes, we show that their volatility has not only increased over time but was also higher during the COVID-19 pandemic. Dynamic unconditional correlation analysis shows that correlations between asset pairs increased during the pandemic, and optimized portfolio weights (in a two-asset portfolio) offer less returns on average when correlations are high. We also show evidence of flight to quality, with investors switching from riskier assets (such as conventional and Islamic stocks) to less risky assets (such as sukuk and green sukuk). This evidence is immensely strong during the COVID-19 pandemic phase.

Our work suggests multiple directions for future research. First, asset correlations have clearly changed during the pandemic phase. It will be interesting to explore how these correlations have implications for dynamic trading strategies such as those of a mean–variance investor who tracks forecasted returns given an information set. It is also clear that the information set used to predict asset prices has also been influenced by the pandemic. Second, we have used a Granger causality test to deduce evidence of flight to quality. Future research should build on this by exploring other approaches and methods to exploring flight to quality in light of the pandemic.

## Footnotes

1 For a literature survey on COVID-19, see Sha and Sharma (2020) and Sharma and Sha (2021).

2 Green investments are investments in asset classes, which are aligned with a commitment to the promotion of environmentally friendly business practices and the conservation of natural resources. Green stocks are equities of environmentally friendly companies while green debt instruments are a type of fixed-income instrument that are specifically earmarked to raise money for climate and environmental project.

3 Several studies on equities have explored the unique characteristics of Islamic equities (see, inter alia, Rizvi and Arshad 2018; Rana and Akhter 2015) and for debt instruments (Sukuk) (see, for instance, Azmat et al. 2017; Naifar and Hammoudeh 2016; Önder 2016).

4 Several studies on COVID-19 have shown how the pandemic has influenced and shaped economic and financial relationship between and within a country and amongst groups of countries such as those belonging to regions. For a partial list of studies, see Prabheesh (2020), who examines stock and foreign portfolio investments during pandemic for India; Gil-Alana and Claudio-Quiroga (2020) evaluate the response of Asian stock markets to the pandemic; Yan and Qian (2020) and He et al. (2020) evaluate the reaction of the Chinese stock market to the pandemic; Liu et al. (2020) test the oil-stock returns nexus for the US; Xu (2020) studies stock returns of USA and Canada; Djurovic et al. (2020) study affect on Montenegro, while Sergi et al. (2021) and Ashraf (2020) consider this relation for 76 and 43 countries, respectively; So et al. (2020) examine the case of Hong Kong stock returns; and Salisu et al. (2020) focus on the OECD countries. In related work, Haroon and Rizvi (2020b) show how financial market liquidity is impacted by the pandemic. See also Narayan, Devpura and Wang (2020) and Wei et al (2020) for the exchange rate-COVID-19 analysis.

5 One of the most influential strands of the COVID-19 literature relates to the energy sector; see, for instance, Ertuğrul et al. (2020) who find evidence of a high volatility pattern of the Turkish diesel market during COVID-19. Polemis and Soursou (2020) find that the pandemic influenced stock returns of the Greek energy firms. Akhtaruzzaman et al. (2020) disclose COVID-19 as a moderator to the oil price shock. Gharib et al. (2020) find contagion effect of bubbles in oil and gold markets during the COVID-19 pandemic. Amar et al. (2020) show spillovers of commodity and stock prices in oil producing and consuming countries during the COVID-19 period.

6 Green sukuk and bond initiative of the Indonesian government is part of efforts to reduce greenhouse gas emissions. Initiated in 2018, the green sukuk issuance raised $1.25 billion to finance environment friendly infrastructural projects across Indonesia.

7 https://www.who.int/news/item/27-04-2020-who-timeline---covid-19

8 https://covid19.who.int/region/searo/country/id

9 https://covid19.who.int/region/searo/country/id

10 Granger Causality test has been used to understand and measure flight to quality in the literature where complete set of data is limited; see, for instance, Soylu, & Güloğlu, (2019), and Corsi, Lillo, Pirino, & Trapin (2018), & Sarwar (2017).
